# Radiographic geometry and clinical glenohumeral range of motion after reverse shoulder athroplasty, a retrospective cohort study

**DOI:** 10.1016/j.jor.2021.05.018

**Published:** 2021-05-24

**Authors:** Kaisa Lehtimäki, Jenni Harjula, Joonas Uurinmäki, Juha Kukkonen, Eliisa Löyttyniemi, Jari Mokka, Hannu Tiusanen, Ville Äärimaa

**Affiliations:** a Department of Orthopedics and Traumatology, Turku University Hospital and University of Turku, Turku, Finland; b Primary Health Care Center of Kimito, Finland; c Department of Biostatistics, University of Turku, Turku, Finland; d Coxa Hospital for Joint Replacement, Tampere, Finland

**Keywords:** Reverse shoulder arthroplasty, Range of motion, Retrospective cohort, Radiographic geometry

## Abstract

**Background:**

The range of motion (ROM) in reverse shoulder arthroplasty (RSA), is mechanically limited by the surrounding bony obstacles especially in abduction and rotation planes. However, the clinical effect of implant positioning, prosthesis design, and individual differences in bone morphology, on ROM is obscure. The aim of this study was to investigate the correlation between radiographic geometry and clinical glenohumeral (GH) ROM after RSA.

**Methods:**

RSA patients operated at Turku University Hospital during 2007–2013 were called for radiological and clinical follow-up. Pre- and postoperative true anteroposterior radiographs were obtained and the positioning of the center of rotation (COR) in relation to the surrounding bony structures was measured. Active and passive shoulder and GH abduction, flexion, internal and external rotation ROM were measured with goniometer. The Constant score (CS) and pain visual analogue scale (VAS) were recorded. The correlation between the radiographically measured parameters and the active and passive ROM and clinical outcome was statistically analyzed.

**Results:**

91 shoulders were available for analyses with a mean follow-up of 38.7 months ± SD 20 (range 12–83) months. 77% of the patients were female, the mean age was 73 (SD 9) years. The mean angle between the line of supraspinatus fossa, and the line between COR and lateral edge of the acromion (α-angle) was 127° (SD 14) and the mean angle between the lines from lateral edge of the acromion to COR, and from there to the superior edge of the greater tubercle (β-angle) was 54° (SD 11). The mean active shoulder flexion at follow-up was 118° (SD 26), abduction 104° (SD 32), external rotation 41° (SD 22), internal rotation 77° (SD 21). The mean passive GH flexion was 80° (SD 19), abduction 67° (SD 15), external rotation 31° (SD 16) and internal rotation 34° (SD 14). The mean Constant score at follow-up was 53 (SD 18) and pain VAS 2 (SD 3). The positioning of the radiographically measured COR did not statistically significantly correlate with the ROM or clinical outcome scores.

**Conclusions:**

Postoperative radiographically measured two-dimensional geometry and positioning of the COR does not significantly correlate with the glenohumeral range of motion or clinical results after RSA.

**Level of evidence:**

Level 3, retrospective cohort study

## 1 Introduction

The original concept of reverse shoulder arthroplasty (RSA) by MD Paul Grammont was designed to increase the abduction lever arm and strength of the deltoid muscle in case of cuff tear arthropathy (CTA) by medializing the glenohumeral (GH) center of rotation (COR).^7^^,^^25^ Numerous reports of good clinical functional outcome have since then corroborated this concept, and increased the popularity of RSA worldwide.^5^^,^^12^ However, the circumferential rather than rotational glenohumeral range of motion (ROM) in RSA, is mechanically limited by the surrounding bony scapular obstacles compromising joint mobility especially the abduction and rotation directions.^8^^,^^9^

Increased focus on positioning of the components and various new implant designs with modifications to eg. glenosphere size, distalization and lateralization of the gliding surfaces, have been introduced to improve the free ROM compared to the original Grammont style RSA implant.^8^^,^^11^^,^^24^^,^^26^ Furthermore, ROM in RSA has been studied in virtual and cadaver modelling to better understand the relationship between RSA bony geometry and ROM.^3^^,^^8^^,^^15^, ^16^, ^17^^,^^21^ In a simplistic theory, the ROM of the humerus around the glenosphere is dependent on the obstacle free sector around the COR of the RSA. However, there exists enormous variability in anatomy and physiology between individuals, and it is not clear whether the different designs or theoretical geometric considerations are applicable to determine ROM in a clinical setting.^21^

The purpose of this study was to investigate the correlation of radiographic geometric variables and clinical glenohumeral ROM. The hypothesis was that the radiographically measured two-dimensional obstacle free sector around the COR would significantly correlate with post-operative GH ROM and the clinical results after RSA.

## 2 Materials and methods

**Fig. 1:**
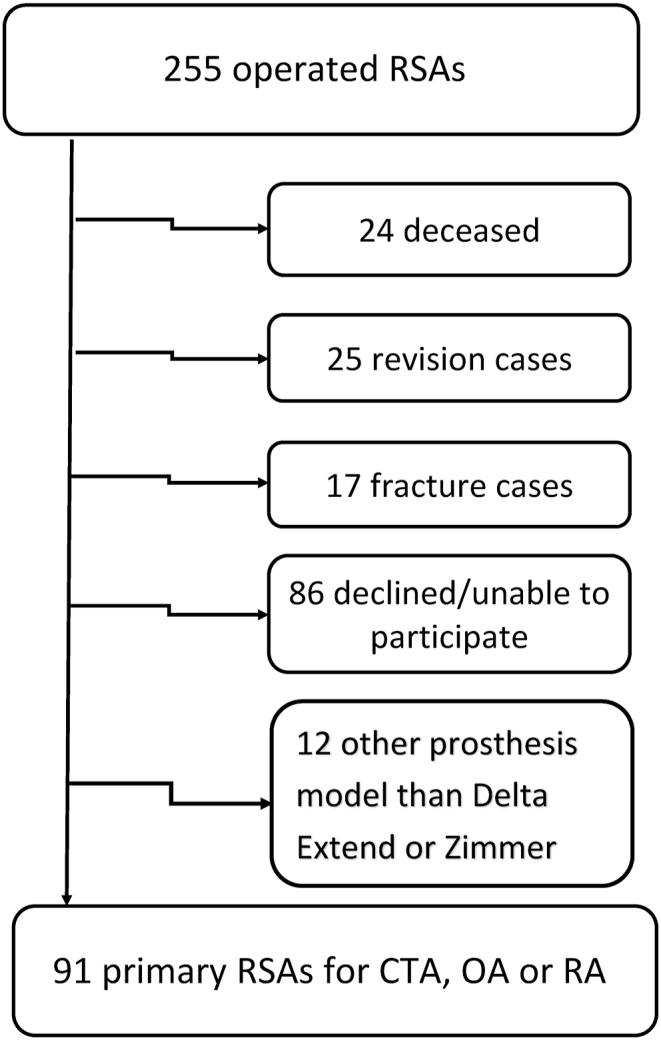
Flow chart.

Approval from the department of Orthopedics and Traumatology at Turku University Hospital was obtained to conduct this study. All patient files containing operational codes for total shoulder arthroplasty at Turku University Hospital during 2007–2013 were obtained. Consecutive patients operated with primary RSA for cuff tear arthropathy (CTA), osteoarthritis (OA) or rheumatoid arthritis (RA), using two mechanically different prosthesis designs: Delta Xtend (DePuy Synthes, Warsaw, IN, USA) with a 155° neck-shaft angle, and Zimmer Biomet trabecular metal TM reverse (Biomet, Warsaw, Indiana) with a 150° neck-shaft angle were included in the analyses. Flow chart is presented in Fig. 1.

Operations were performed by 8 specialized shoulder surgeons. Patients were under general anesthesia, and either a lateral deltasplit or an anterior deltopectoral approach was used. In case of deltopectoral approach the subscapularis tendon was detached from the humerus to gain exposure. The humeral head and osteophytes were resected and a full release around the glenoid was performed. The metaglene, glenosphere, and humeral components together with polyethylene inserts were attached according to surgeon preference. The subscapularis was reattached if possible and the arm was immobilized in a sling after the wound closure. Passive ROM exercises were commenced after three weeks and active training six weeks after the operation.

**Fig. 2:**
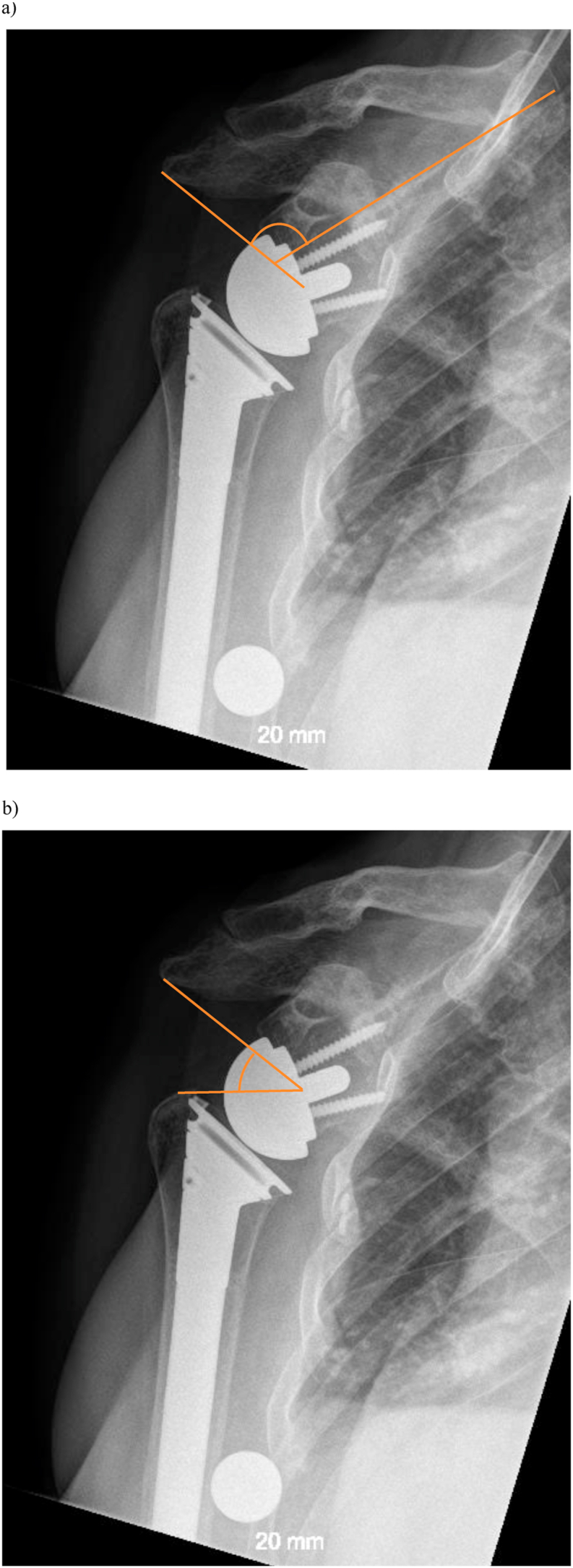
**a.** the α-angle is the angle between the line from supraspinatus fossa and lateral aspect of the acromion to the COR. **b.** the β-angle is the angle between the line lateral aspect of the acromion and the line from lateral aspect of the tuberculm major to the COR.

All patients were called for radiographic and clinical and follow up. True anteroposterior plain radiographs (30-degree oblique view) in standing position (arm in neutral rotation and 0° of abduction) were obtained preoperatively, immediately postoperatively, and at follow-up. The preoperative images were analyzed for cuff tear arthropathy according to Hamada,^10^ and critical shoulder angle (CSA).^19^ Images taken immediately postoperatively and at follow-up were analyzed specifically for scapular geometry around the COR of the RSA. The main focus was on the angle between the line of supraspinatus fossa, and the line between COR and lateral edge of the acromion (α-angle), the angle between the lines from lateral edge of the acromion to COR, and from there to the superior edge of the greater tubercle (β-angle) (Fig. 2). In addition parameters such as previously described lateralization angle (LSA), the distalization angle (DSA),^4^ and the glenosphere inferior offset from inferior glenoid (GIO) were measured. At follow-up signs of notching were analyzed according to Sirveaux.^1^^,^^23^

The active and passive shoulder ROM was measured in a standing position using a goniometer. Passive glenohumeral abduction and flexion ROM was measured while stabilizing the scapula with one hand and moving the humerus with the other, while the assistant held the goniometer. The rotational passive ROM was measured in a supine position with humerus in 60° of abduction. The Constant score (CS), Western Ontario Osteoarthritis Score (WOOS), and pain visual analogue scale (VAS) were recorded as measures of clinical outcome.

Continuous variables are summarized with mean and standard deviation (SD). The glenosphere size adjusted Pearson correlation (partial correlation) between the radiographically measured parameters and the active and passive ROM and clinical outcome was calculated. Statistical significance level was set at p = 0.05 (two-tailed). The analyses were performed using SAS software, version 9.4 for Windows (SAS Institute Inc., Cary, NC, USA).

## 3 Results

Altogether 91 shoulders with primary RSA for CTA (n = 48), OA (n = 10), or RA n = 28 were available for analyses. There were 47 Delta Xtend and 44 Zimmer Biomet trabecular metal prosthesis and the mean glenosphere size was 39 mm (SD 2.6), only centric glenosphere components were used. The mean age of the patients was 73 years (SD 9) and 77% of them were female (Table 1). The mean follow-up was 38.7 months (SD 20; range 12–83).

**Table 1:** Patient demographics.

Variable Data
Gender Female/Male (n) 69/20
Mean BMI 27 (SD 6)
Mean age at the surgery (years) 73 (SD 9)
Mean follow-up time (months) 38.7 (SD 20)
Indication for surgery
-cuffartropathy 48 (53%)
-rheumatoid arthritis 28 (31%)
-osteoarthrosis 15 (16%)
Implant brand
-Delta Extend 47 (51,6%)
-Zimmer TM 44 (48,4%)

**Table 2:** Mean clinical outcomes at follow up.

VAS 2.2 (SD 2.6)
Constant score 53 (SD 18)
WOOS index 67.2 (SD 24)
active flexion 118° (SD 26)
active abduction 104° (SD 32)
active external rotation 41° (SD 22)
active internal rotation 77° (SD 21)
passive glenohumeral flexion 80° (SD 18)
passive glenohumeral abduction 67° (SD 15)
passive glenohumeral external rotation 31° (SD 16)
passive glenohumeral internal rotation 34° (SD 14)

Preoperatively the radiological mean Hamada grading was 3 (SD 1.4, range 0–5). The mean CSA was 37° (SD 7, range 20–55). Post-operatively the mean α-angle was 127° (SD 14, range 94–168), and β-angle 54° (SD 11, range 19–80). The LSA and DSA angles were 81° (SD 15, range 39–132) and 52° (SD 13, range 16–97), respectively. The mean immediate postoperative GIO was 3 mm (SD 3, range 3). There was detected above grade 2 Sirveaux notching in 16 (18%) cases at follow up (grade 0 in 23, grade 1 in 35, grade 2 in 14, grade 3 in 11, and grade 4 in 5 cases). There were 9 shoulders with radiologic downward tilted acromion stress fracture at follow-up .

At follow up the mean active shoulder flexion was 118° (SD 26), abduction 104° (SD 32), external rotation 41° (SD 22), and internal rotation 77° (SD 21) degrees. Accordingly, the mean passive flexion 135° (SD 19), abduction 111° (SD 21), external rotation 54° (SD 24), and internal rotation 89° (SD 21) degrees. The mean passive GH flexion with a fixed scapula was 80° (SD 18), abduction 67° (SD 15), external rotation 31° (SD 16) and internal rotation 34° (SD 14). The mean CS was 53 (SD 18), WOOS index 67.20 (SD 24) and VAS 2 (SD 3) at follow-up (Table 2).

There was a statistically significant correlation between the preoperatively radiographically measured CSA and post-operative α-angle and, also LSA and DSA angles (Table 3). However, there was no statistically significant correlation between the measured glenosphere size adjusted geometrical parameters and ROM or clinical outcome. The measured glenosphere inferior offset did not significantly correlate with the detected notching (r = 0.17, p = 0.1518). However, notching was statistically significantly correlated with the measured α-angle (r = 0.38, p 0.0013) and preoperative CSA (r = 0.34, p = 0.008).

**Table 3:** Mean values and Pearson's correlation coefficients of radiographic geometric parameters and measured α-angle.

α-angle (mean)	127° (SD 14)	–
β-angle (mean)	54° (SD 11)	r = −0.49 (p < 0.0001)
CSA (mean)	37° (SD 7)	r = 0.50 (p = 0.0001)
LSA (mean)	81° (SD 15)	r = 0.36 (p = 0.0008)
DSA (mean)	52° (SD 13)	r = −70 (p < 0.0001)

## 4 Discussion

The main finding of this study was that none of the radiographically measured geometrical parameters were statistically significantly associated with the clinical ROM nor post-operative outcome scores. This is in accordance with the previously reported findings by Robertson et al.^21^ The clinical results of RSA in our series were comparable to the published literature.^1^^,^^2^^,^^14^

The COR based α-angle in our study statistically significantly correlated with the previously reported CSA angle. This implies that the preoperatively measured scapular morphology ie. CSA angle determines the positioning of COR in RSA, and on the other hand that the glenosphere implants in our series were systematically positioned in relation to the native glenoid. Despite the pre- and postoperatively detected variability in the radiographic geometry, we did not find a statistically significant correlation between measured positioning of RSA COR (α-angle) and active shoulder ROM, and moreover with the passive GH ROM with a fixed scapula.

The α-angle is dependent on both the lateralization and distalization of the COR. However, it does not take into account the positioning of the humerus in relation to the COR. A large glenosphere pushes the humerus distally away from the scapula and there are previous reports of a positive correlation between the glenosphere size and ROM.^18^^,^^20^^,^^26^ Therefore we adjusted the statistical analyses according to the glenosphere size. Moreover, we measured also the β-angle, ie. the free sector between the acromion and the humeral tubercle at 0° of abduction. We could neither find a significant correlation between the β-angle and active or passive ROM. Contrary to our findings, radiographic geometry has been previously reported to influence ROM in RSA.^4^^,^^6^ Especially the LSA allegedly positively correlates with both postoperative active ROM and also CS, although this correlation could be regarded as weak and arbitrary.^4^ Nevertheless, the LSA and DSA angles do not take into account the COR, which in our opinion should be the key interest while planning for RSA. In clinical practise it would be very helpful to have a clinically relevant target position for the COR of implants to optimize the outcome of RSA. The radiographic geometric parameters used in this study were in good agreement with each other, i.e. similarly representing the anatomy, however all insufficient to provide a statistically significant clinical relevance for ROM in RSA. It is plausible that e.g. the variable positioning and functionality of the scapula, and on the other hand the soft tissues outweigh the influence of GH bony geometry on shoulder, and passive GH ROM, respectively. In any case we could not reject our null hypothesis as we found no significant correlation between the positioning of the COR and ROM.

Previously Helmkamp et al. reported that prosthetically lateralized COR yields in increased postoperative external rotation and decreased scapular notching.^13^ We did not find significant differences in ROM or notching between the prosthesis models. Interestingly, there was a positive and statistically significant correlation between the CSA and alpha angle and notching at follow-up. This finding was also independent from the glenosphere size and inferior offset. A pronounced acromion overhang may be associated with other unknown factors, eg. short glenoid neck, potentially contributing to the increased risk of notching. Nevertheless, similarly to previous reports notching was not associated with ROM or clinical outcome in our study.

Our retrospective analyses of consecutive primary RSAs has several limitations. Firstly, we lost many patients to follow-up mainly due to general health related problems. Secondly, we used only one plane two-dimensional imaging, and only one experienced observer to measure the radiographic parameters. However, in previous reports both intra- and interrater reliability of these type of measurements have been found to be very good.^4^ Thirdly, we do not have accurate data on the preoperative nor perioperative functional status of the patients. It has been previously reported that the perioperative ROM is strongly associated with the ROM at follow-up.^22^ Fourthly, we did not assess the active scapular kinesiology, nor for condition of the muscles around the glenohumeral girdle. Fifth the patient population was very heterogenic with multiple potentially confounding factors. On the other hand our study represents a true clinical consecutive patient population.

## 5 Conclusion

Postoperative two-dimensional radiographically measured obstacle free sector around the COR does not significantly correlate with shoulder nor glenohumeral range of motion or clinical results after RSA. More research and more detailed imaging is needed together with analyses of other potential physical and physiological factors to evaluate the prognostic factors for range of motion and clinical outcome in RSA.

## Authors’ contribution

Kaisa Lehtimäki: study design, data collection and manuscript preparation. Jenni Harjula: data collection. Joonas Uurinmäki: data collection. Juha Kukkonen: manuscript preparation. Eliisa Löyttyniemi: biostatistics. Jari Mokka: data collection. Hannu Tiusanen: data collection. Ville Äärimaa: study design and manuscript preparation

## Disclosure of interest

The authors declare that they have no competing interest.

## Sources of funding

The research was funded by personal grant for the corresponding author by Finnish Arthroplasty Association and Research Foundation for Orthopaedics and Traumatology

None of the authors or a member of their immediate family had any financial bias regarding this manuscript.

Approval from the department of Orthopedics and Traumatology at Turku University Hospital was obtained to conduct this study
